# Needs to address clinicians’ moral distress in treating unvaccinated COVID-19 patients

**DOI:** 10.1186/s12910-022-00859-9

**Published:** 2022-11-14

**Authors:** Robert Klitzman

**Affiliations:** Columbia University, 1051 Riverside Drive, Mail Unit #15, New York, NY 10032 USA

**Keywords:** Medical ethics, Medical professionalism, COVID-19, Triage, Moral conflict, Moral distress, Provider mental health, Medical education, Policy

## Abstract

**Background:**

Moral dilemmas have arisen concerning whether physicians and other providers should treat patients who have declined COVID vaccination and are now sick with this disease. Several ethicists have argued that clinicians have obligations to treat such patients, yet providing care to these patients has distressed clinicians, who have at times declined to do so. Critical questions thus emerge regarding how best to proceed.

**Main body:**

Providers face moral tensions: whether to place the benefits to an unvaccinated patient over their duties to protect themselves and their families, staff and other patients, and goals of working collaboratively with patients. Clinicians’ duties to treat such patients arguably outweigh claims otherwise, but these obligations are creating moral conflict and distress for providers. Moral distress has been associated with burnout, post-traumatic stress disorder, and interpersonal and work difficulties. Given ongoing vaccine refusals, these problems are unlikely to disappear in the foreseeable future. Society has obligations to address this moral distress due to principles of reciprocity, and implicit social contracts, as part of which physicians risk their lives in caring for patients for the good of society as a whole. Responses are thus urgently needed at several levels: by hospitals, medical schools, professional societies, governments, media, providers and patients. Medical training on professionalism should address these stresses, probing why doctors have duties to treat these patients, but also how moral conflicts can ensue, and how best to address these tensions. Governments and institutions should thus alter relevant policies and devote more resources to addressing clinicians’ psychological strains. Institutions should also improve organizational culture. Public health organizations and the media described clinicians, earlier in the pandemic, as heroes, committed to treating COVID patients. This narrative should now be changed to highlight the strains that unvaccinated patients cause—endangering hospital staff and others.

**Conclusions:**

Unvaccinated COVID patients should receive care, but multi-level strategies, involving enhanced policies, education and practice are vital to alleviate ensuing moral distress, and thus aid these clinicians and their patients. Ethical arguments that providers must treat these patients have not considered these obligations’ effects on clinicians, but should do so.

## Background

Unvaccinated COVID-19 patients in many countries have been using limited resources, such as intensive care unit (ICU) beds, causing moral distress among front-line clinicians in ways that urgently need to be addressed. Multiple surges of the COVID pandemic have occurred; and though a recent increase, caused by the Omicron variant, has subsided in many regions, leading epidemiologists anticipate that additional such surges of this and other new viruses will occur [1]. Vaccination and other public health measures (e.g., masking, testing, contact precautions, social distancing) need to be encouraged at all levels, but have faced resistance from large proportions of the population. In the U.S. and several industrialized nations, most adults have been fully inoculated, but sizable proportions, often a third, have not [2]. Unvaccinated patients who develop serious COVID have thus crowded hospitals, reducing staff-patient ratios, jeopardizing themselves, their families, friends and healthcare professionals, impeding treatment of other, non-COVID patients, and posing critical ethical questions. The fact that the monoclonal antibody treatment Paxlovid has been found to be more efficacious against COVID among unvaccinated individuals than vaccinated patients has exacerbated these tensions [3].

Questions regarding allocations of scarce resources have received attention since the start of the COVID pandemic, initially with regard to distributing ventilators [4], yet the wide availability of vaccines that many severely ill patients refuse to take raises additional questions.

Specifically, heated controversies have arisen over whether physicians are obligated to treat COVID patients who have chosen not to be vaccinated, and if so, why or why not, and how this moral dilemma should be addressed.

## Main text

### Resistance to treating unvaccinated COVID-19 patients

Several ethicists have argued that providers have an obligation to treat unvaccinated patients [5–7], and that COVID vaccine status should not be used in tie-breaking in allocation of scarce resources [8]. In determining which patients should and should not receive such resources during the pandemic, ethicists have widely drawn on principles of triage [9], developed in wartime situations, when soldiers get wounded in performing their duty for the benefit of their country (i.e., not if they purposefully walk into the line of fire). These principles dictate that such limited resources be distributed based on assessments of which patients are most likely to benefit and most likely to suffer without these interventions, regardless of diagnosis alone per se [10]. Physicians might then  afterwards use secondary considerations if needed, prioritizing, for example, front-line clinicians who then can treat additional patients. Yet unvaccinated patients may be the most in need of scarce resources, and the most likely to benefit from these, and thus justified in receiving these resources, despite their decision not to get vaccinated.

Considerable discomfort among clinicians has arisen, however, along with debates about needs to treat these patients. Various U.S. providers have in fact expressed concerns to supervisors and each other and at times declined to provide such care. Doctors in Alabama, Florida, the District of Columbia and Toronto have refused to see unvaccinated patients [11]. A North Texas task force wrote a memo that supported denying ICU beds to unvaccinated patients, though after the memo received media attention, the task force announced that it was reversing its decision [12]. A recent prominent *Washington Post* op-ed entitled, “Doctors should be allowed to give priority to vaccinated patients when resources are scarce” [13] argued that, “vaccinated patients should be given priority over those who have refused vaccination without a legitimate medical or religious reason,” and that, “it would be morally wrong *not* to give priority care to the heart attack victim” over an unvaccinated COVID patient [13].

Arthur Caplan, a prominent bioethicist, also recently argued that in primary care, physicians have the right not to accept patients who refuse to get these vaccines, since such patients are thereby refusing to follow that physician’s advice, and are endangering the doctor, other medical staff and other patients in the waiting room [14]. This article clarified that this right to deny unvaccinated COVID patients care did not extend to ICUs and emergency rooms, where patients are severely ill, but that physicians could potentially use vaccine refusal as a reason to deny certain treatments, if non-vaccination predicts unsuccessful treatment response. Nonetheless, this online article generated many comments that averred that doctors should in fact treat all unimmunized patients [14].

### Reasons against treating unvaccinated patients

In confronting requests to treat unvaccinated patients, providers are facing moral tensions. On the one hand, physicians, based on the principles of beneficence, have obligations to society to aid other, current and future patients, and to protect co-workers (who care for additional patients), and thus to remain healthy and able to work. If physicians treat unvaccinated patients, other, vulnerable patients may then be at increased risk for exposure. Physicians must thus weigh a patient's rights to refuse vaccination against their own duties to reduce risks, including such a patient’s potential harm to other patients (e.g., in the ER, hospital and doctor's waiting room). Such other patients may be immunocompromised or have pre-existing conditions, and/or may be elderly and/or people of color, and hence be more vulnerable to serious symptoms if they become COVID-infected. Doctors also have moral obligations to society, which has provided resources for their education and training.  From a utilitarian perspective, a doctor should help as many patients as possible, and therefore steward current and future use of limited resources, including his or her own time.

Staff and patients, even if vaccinated, should still wear masks in all hospitals, physicians’ offices, and waiting rooms, lowering risks of infection from any infected patients who might be present. Yet, personal protection equipment (PPE) is not 100% effective. Consistent use of N95 masks lowers infection rates by around 44%, not 100% [15], and many patients prefer to wear less effective surgical masks [16]. Moreover, patients and their families and staff may occasionally need to remove these facial coverings to eat or drink. Unvaccinated patients could therefore still endanger other patients, family members and staff in ERs, hospitals or a doctors’ waiting rooms.

Physicians, as individuals, spouses and parents also have rights and duties to keep safe and protect themselves and their families.

In addition, patients who refuse vaccinations appear to be breaking an implicit social contract between providers and themselves: that “doctors care for patients” and “patients care for themselves and others.” Patients have not only rights, but responsibilities as well—to make responsible decisions about their own care, cooperate with treatment plans, and pursue self-care, given their relational responsibilities to others in their lives [17, 18]. Sociologists have described the “sick role,” in which society excuses patients from certain obligations, such as working, but in return, requires that these individuals do all they can to get better [19]. Furthermore, patients who refuse vaccination endanger not only their own health, but that of others in a wide range of social settings (e.g., with family members, co-workers, fellow store customers and bus and subway passengers), and therefore have high likelihoods of contributing to the ongoing spread of the pandemic and potentially the evolution of new variants, all of which can endanger public health, and further strain hospitals and healthcare systems that have already been severely stressed. While a small minority of unvaccinated patients may have medical reasons for their refusal (e.g., past adverse medical reaction to components in the vaccine), the vast majority of refusers do not. Patients who now continue to refuse vaccination without having such a medical reason are thereby failing to sufficiently appreciate or value the risk that they then pose to others, disregarding crucial norms of beneficence, and implicit social contracts and reciprocity with medical providers. A patient refusing vaccine may therefore undermine providers’ sense of responsibility to uphold their own sides of such social contracts. Physicians and other medical staff may therefore feel less obligation to treat such patients, since this duty stems partly from this implicit social contract. In the current polarized political climate, fueled by social media, COVID unvaccinated patients have also at times directed vitriol at healthcare and public health professionals [20], further angering, frustrating and distressing providers.

As part of the patient/clinician relationship, a provider should ask patients who have declined vaccination why they have done so. Yet since vaccination has become highly politicized—e.g., with many of former President Trump’s supporters strongly opposing it [21], patients may not want to admit that they are refusing vaccination for such political reasons, and may instead cite religious or philosophical objections. Still, no major national leader of a religion has opposed vaccines [22]—even Pope Francis I has urged all Catholics to get vaccinated [23]. Physicians’ efforts to elucidate reasons for vaccine refusal and to address these barriers and persuade patients otherwise may thus not always succeed.

### Reasons to treat unvaccinated patients

Yet despite these arguments against offering treatment, strong deontological as well as consequentialist arguments support physicians instead caring for unvaccinated patients. Patients have vital rights of autonomy to decide about their own treatment, and hence to refuse vaccination, if they wish, and physicians have strong professional and moral duties to beneficence to care for patients, even if the latter are unvaccinated. Professionalism includes notions of altruism, compassion, unselfishness, helping others, placing patients’ interests above one’s own, being trustworthy, avoiding conflicts of interest and upholding responsibilities to the profession [24, 25]. In entering their profession, physicians recognize and accept that doing so and administering to the sick may at times entail physical risks to themselves and their families.

Clinical care depends, too, on trust—on patients’ confidence that they will not be judged for potentially stigmatized behaviors or decisions. Doctors and nurses therefore have responsibilities not to judge patients for unhealthy behaviors. Clinicians routinely treat patients with emphysema due to smoking, diabetes from overeating, cirrhosis from alcohol abuse, and accidents from not wearing seatbelts, or engaging in other risky behaviors that endanger the individual and at times others and are hence costly to the healthcare system and society.

Treatment of such unvaccinated COVID patients by a physician is further justified since it may decrease the risk these patients may otherwise, if untreated, pose to others. In addition, a doctor could also reduce risks from unvaccinated parties by treating them in a dedicated examination room to reduce the potential dangers to other patients.

While arguments have been made that certain private primary care physicians may have a right to refuse to treat unvaccinated patients [13], patients in many primary care settings may lack choices of instead going to other clinics or doctors for care, because of limitations of insurance or geography. Patients may also have long-standing, ongoing relationships with these clinicians and institutions that could be challenging to sever, for both providers (because of professionalism) and patients.

Exceptions to these needs to provide care might exist when a particular treatment is in very short supply and vaccination is essential for survival. Notably, with organ transplantation, for instance, patients who refuse immunization are given lower priority, since organ recipients must undergo immunocompromise to avoid organ rejection, and consequently have much heightened risks of morbidity and mortality if they become COVID-infected, justifying requirements for vaccination in this situation [26].

Logistical problems would arise as well in trying to incorporate vaccine status into triage decisions. While *The Washington Post* article, for instance, proposed to deny care to COVID patients who refused vaccination, “without a legitimate medical or religious reason” [13], doctors usually don’t know *why* a patient has avoided vaccination, and refusers may simply claim that their reasons are religious when that is not the case. Patients may have believed disinformation from social media or members of their community, or had real or perceived medical concerns, or limited access to vaccines. Others may hesitate because of long-standing ethnic or racial discrimination by medical institutions. Roles of vaccination among African-Americans, though increasing, still remain lower than for Caucasians [2]. From a justice perspective, deprioritizing non-vaccinated individuals would thus worsen current healthcare disparities and heighten mistrust among certain groups. Patients also often significantly misunderstand the needs for vaccines. Severely ill COVID patients have told colleagues, for instance, “I’m now willing to get a vaccine,” not comprehending that it is too late now to do so to prevent serious COVID. Providers and public officials in public health and other areas should emphasize that science unequivocally demonstrate that vaccines are extremely effective.

### Needs to treat resultant moral distress

Physicians’ duties to treat unvaccinated patients arguably thus outweigh arguments otherwise; but importantly, these obligations are causing moral conflict and distress among providers, and not all doctors feel fully persuaded by these arguments to provide treatment. Prior discussions of treatment that clinicians should provide, rather than deny to these patients [10] have, however, not examined these complexities and moral strains, which urgently need to be recognized and addressed.

Moral distress occurs when providers feel forced to act contrary to their values [27] and unable to “preserve all interests and values at stake” [28]. Moral distress has been examined among nurses who are instructed by a physician to carry out a treatment that they themselves feel will harm patients [29]. In these situations, nurses are forced to place institutional needs to adhere to a subordinate role over their own perceptions of how to most benefit the patient, creating conflict of values [6].

The moral distress providers now feel caring for unvaccinated patients differs in certain regards from these other situations. With unvaccinated COVID patients, providers experience tension because these professionals could themselves get infected, making them unable to treat other patients, and exposing their own families as well as other patients. These professionals thus face moral conflicts between their obligations to treat every patient (regardless of the patient’s vaccination decisions) and their duties to protect themselves and their family, staff and other patients, and goals of working collaboratively with patients.

For clinicians, treating unvaccinated COVID patients creates additional moral strains that do not occur in caring, for instance, smokers and alcoholics, who are also endangering themselves and incurring healthcare costs, but are not also directly jeopardizing others by spreading COVID, and thereby exposing clinicians and leading to more cases. Seriously ill unvaccinated COVID patients are also using scarce resources, such as ICU beds, in ways that smokers and drinkers usually do not.

Medical staff have thus argued that unvaccinated COVID patients should be considered differently because they threaten providers themselves. Similarly, in the early days of the AIDS pandemic, many medical trainees did not want to work in regions with high HIV prevalence, and chose training programs in other regions of the country [30]. But historically, doctors have at times had to face risks in treating various infectious diseases. Doing so can, however, nonetheless generate significant strains.

These difficulties can thus cause and/or exacerbate moral and psychological distress and burn out. Due to COVID, burnout among physicians is at an all-time high, with 63% of physicians reporting at least one symptom of it [31]. Since the pandemic began, physicians have struggled, witnessing the deaths of countless patients and co-workers, often due to patients who have not taken precautions such as wearing masks and quarantining when sick. Earlier in the pandemic, PPE was not sufficiently available, and many providers and their colleagues became ill [32]. Physician burnout was rising even prior to the pandemic and vaccine roll-out [33], with almost half of physicians (45.8%) experiencing symptoms [34]. COVID has increased such stresses. After a year of the pandemic, and even before the development of vaccines and of COVID patients declining such immunization, clinicians endured strains, rationing PPE, ventilators, ICU beds, and staff, and seeing many patients dying. Ratios of patients to nurses have increased two- to four-fold [35, 36]. With COVID, almost a third of healthcare workers have felt stressed and depressed, and considered leaving the profession, worsening staff shortages, especially of nurses [27, 37]. Rates of burnout among hospital staff have increased about 62%—from 27% to 44.2% [38]. Moral distress has been associated with burnout, post-traumatic stress disorder symptoms, and interpersonal and work difficulties [39]. Failure to consider and address this moral distress can exacerbate problems since heightened provider burnout can threaten the quality of care delivered, and thus health outcomes.

Detailed quantitative studies measuring the precise amounts of physician moral distress resulting from unvaccinated patients have not yet been published, given the relative newness of the vaccine roll-out, and the delays that can exist in obtaining institutional review board (IRB) approval for conducting studies, preparing manuscripts and responding to journal reviewers and in publication—all of which can easily take over a year—with additional time elapsing if the researchers also first need to apply for government or other funding to conduct the research. However, the fact that approximately one-third of the population in the U.S. and other Western countries has not yet been fully vaccinated (despite the wide availability of vaccines), which has further fueled the pandemic (with approximately 1.7 million cases per month in the U.S. and 635 million in the world as of November 11, 2022 [40]), and major news outlets (e.g., *The Washington Post*) and some bioethicists supporting physicians’ hesitancy to treat unvaccinated patients in at least certain situations, all highlight the breadth of the problem and needs for concern [13, 14].

### Ways of addressing resultant moral distress

Given possibilities of the COVID virus continuing to mutate [41], and millions of people undoubtedly continuing to refuse vaccination, these problems are unlikely to disappear in the foreseeable future.

Suggestions have been made to reduce the numbers of seriously ill unvaccinated COVID patients by charging higher premiums to unvaccinated individuals. At least one employer has also proposed having unvaccinated workers pay a health insurance surcharge [42]. But at least in the U.S., the Kaiser Family Foundation has concluded that the Affordable Care Act and other laws bar insurers from doing so [42]. Such higher premiums could also potentially incentivize vaccination, but unfairly disadvantage certain groups who are wary due to long-standing discrimination.

These clinicians’ moral distress will hence likely continue, and needs to be addressed, rather than simply ignored. Ethically, society is obligated to care for physicians facing moral distress, for both consequentialist and deontological reasons—to ensure ongoing benefit to future patients and therefore society and to meet duties of reciprocity—in return for these physicians risking their own health for the benefit of society as a whole. Implicit social contracts exist between physicians and not only individual patients, but society more broadly. Doctors dedicate themselves to caring for the sick in society in return for certain privileges, including governmental support of medical training, other assistance and in this case, to ameliorate such distress.

To reduce this moral distress, actions are therefore vital at several levels, including those of hospitals, medical schools, professional societies, federal and state governments, providers, patients and the media. These various stakeholders need to grasp and appreciate these moral strains. Arguments that providers should simply treat all unvaccinated COVID patients must consider the unintended consequences. Efforts to increase immunization will help, yet unvaccinated patients will likely continue to get severely ill from COVID and need treatment with scarce resources, stressing providers.

At institutional levels, obligations to treat unvaccinated COVID patients underscore needs to lower staff stress by improving organizational culture, reducing administrative burdens on clinicians (e.g., redundant and unnecessarily burdensome documentation requirements), avoiding increases in hours worked by providers and trainees, optimizing health information technologies, and encouraging and facilitating teamwork [43].

In addition, hospitals should offer adequate resources and sufficient time off, and make mental health and other beneficial services readily available and accessible. During the COVID pandemic, many medical centers held “Employee Appreciation Days” and posted signs thanking staff. These gestures are welcome, but insufficient in and of themselves, and to many staff, feel like “Band-Aids.” Holding memorial services for staff to attend in honor of deceased patients or fellow employees can potentially also help in the short term, reducing burnout from patient deaths [44]. But given the frustration, anger and moral distress from treating unvaccinated patients, policymakers and hospitals should do more.

Some hospitals have provided psychologists or psychiatrists to meet with staff to provide assistance individually or in groups informally, not as formal treatment per se. More institutions should establish such opportunities, if needed, for providers to discuss these tensions and reconnect with the underlying ideals that inspired them to enter their profession. Mindfulness trainings can be beneficial as well, yet given providers’ busy schedules, sustained and ongoing mindfulness can be hard to achieve. Hospitals need to work to make each of these types of activities as easy and feasible as possible for providers to attend—for example, holding such sessions during the time allotted for Grand Rounds, when physicians do not schedule other meetings.

Given ongoing duties to treat unvaccinated patients, medical educators should also be as aware as possible of these challenges and of needs to teach trainees to deal with such patients. Professionalism education often focuses on broad general principles, rather than on what to do when these and other ethical principles conflict and need to be weighed against each other. Education on professionalism should thus address this situation of doctors not wanting to treat unvaccinated COVID patients, probing why doctors nonetheless have duties to care for these patients, how and why psychological and moral conflicts can nonetheless ensue, and how best to address these challenges [45].

Clinicians can benefit, too, from enhanced awareness and discussion of this challenge and their resultant feelings. Providers and trainees need to be aware of, and reflect on, their frustrations with uninoculated patients, rather than simply experiencing and acting on these frustrations without reflection. Providers can develop strong feelings toward patients, known as counter-transference, which can impede the quality of care [46]. Clinicians are obliged not to have warm feelings towards all patients, but to counter any negative feelings, and to therefore recognize, address, and overcome any negative counter-transference feelings they may have. Yet doing so can be psychologically and emotionally difficult and require high psychological awareness or assistance.

Given that the mental health system is already overburdened, federal and state governments also need to devote more resources to addressing these strains among clinicians. At the federal level, the Dr. Lorna Breen Health Care Provider Protection Act, which the U.S. Congress is now considering, would increase awareness and provide grants for programs that offer mental health services for front-line healthcare staff [47, 48]. This bill would provide important benefits, yet other institutional and systemic changes and significant commitment and efforts are vital as well to address the causes of such burnout.

In addition, state medical boards need to alter certain current policies. Though trainees and doctors have high rates of burnout, depression, and substance abuse, and higher rates of suicide than the population at large [49], they avoid seeking treatment because of fears of harms to their career due to several policies. Among medical residents and fellows, for instance, 61% thought they would benefit from treatment, but only 24% sought it, with 76% concerned about confidentiality and 50% worried about difficulties continuing licensure afterwards [50]. Many states ask doctors, as part of state license renewal, whether these professionals have any mental health problems, and answering affirmatively can threaten licensing. The Federation of State Medical Boards has recommended that such questions be changed, to require physicians to report mental health problems *only if* these symptoms impact job performance, and occurred within the past two years, and the physician is neither being monitored nor in good standing in a Physician Health Program. This Federation recommends, too, that state boards use supportive or normalizing language about obtaining mental health treatment. But only one state has thus far adopted all these guidelines [51]. Physicians in states in which initial license applications fail to follow such guidelines are 29% more reluctant to seek mental health treatment [52]. More state boards need to follow these recommendations in order to support doctors in admitting, and seeking treatment for, mental health issues now faced.

Malpractice and disability insurance companies also still regularly ask about any past or current mental health diagnoses and treatment, rather than only current impairments [53], and should alter these policies. Federal and state may need to assist by encouraging or requiring such changes.

Additionally, public health campaigns, policymakers, and the media should shift how they portray and discuss providers. News organizations described professionals, earlier in the pandemic, as heroes, strongly committed to treating COVID patients. The media should now change this narrative to highlight more fully the immense strains that unvaccinated patients cause—endangering hospital staff, loved ones, and the nation as a whole. Public health officials and policymakers should seek further input from experts in communications, media, and advertising to develop such strategies and messages, highlighting more forcefully how much decisions to avoid vaccination are harming and stressing healthcare providers and our country. Such messages might emphasize, for instance, that, especially as the COVID pandemic continues, if people want a doctor to help them when they are sick for any reason, they should get vaccinated, since otherwise, providers may be in ever-shorter supply. The American Medical Association, the Association of American Medical Colleges and other professional organizations can encourage, aid and help guide these efforts as well.

## Conclusions

Moral dilemmas have arisen concerning whether physicians and other providers should treat patients who have declined vaccination and are now sick with COVID, and how to address moral conflicts that ensue. Claims both for and against such treatment can be made. On balance, however, professional obligations to provide care appear paramount. Principles of autonomy support these patients’ rights to make their own decisions about their treatment. Beneficence requires that medical professionals provide care to patients in non-judgmental ways. Non-maleficence requires efforts to avoid further loss of trust among patients who may have declined vaccination because of past discrimination from the medical system. Principles of justice are critical as well, since among vaccine decliners, people of color are disappropriately represented [2]. Clinicians should thus provide rather than refuse treatment to unvaccinated COVID patients.

Yet society also has obligations to address the moral conflict and distress that physicians and other providers confront. Principles of reciprocity, and implicit social contracts (as part of which physicians risk their lives caring for patients for the good of society as a whole) also urgently require heightened efforts to reduce the moral distress clinicians confront. Efforts are critical at several levels—through appropriate institutional and governmental policies, education and practice. Arguments that providers must treat these patients should also consider the effects of these obligations. This need to reduce moral distress does not overturn obligations to provide treatment, but should be addressed at several levels as critical parts of responses to the pandemic. As COVID cases continue, this approach can best aid both vital healthcare professionals and their patients.

## Data Availability

Data sharing is not applicable to this article as no datasets were generated or analyzed.

## Abbreviations

ICU: Intensive care unit
IRB: Institutional Review Board
PPE: Personal Protective Equipment

## References

[1] Jewett C. Fauci predicts an uptick in U.S. cases, saying it is not yet time to ‘declare victory.’ [Internet]. The New York Times; 2022 March 20 [Cited 2022 November 11]. Available from: https://www.nytimes.com/2022/03/20/health/fauci-us-cases-ba2.html?searchResultPosition=1.

[2] Ndugga N, Hill L, Artiga S, Haldar S. Latest data on COVID-19 vaccinations by race/ethnicity [Internet]. KFF; 2022 July 14 [Cited 2022 November 11. Available from: https://www.kff.org/coronavirus-covid-19/issue-brief/latest-data-on-covid-19-=vaccinations-by-race-ethnicity.

[3] U.S. Food & Drug Administration (FDA). Coronavirus (COVID-19) update: FDA authorizes first oral antiviral for treatment of COVID-19 [Internet]. FDA; 2021 December 22 [Cited 2022 November 11]. Available from: https://www.fda.gov/news-events/pressannouncements/coronavirus-covid-19-update-fda-authorizes-first-oral-antiviraltreatment-covid-19.

[4] Mannelli C (2020). Whose life to save? Scarce resources allocation in the COVID-19 outbreak. J Med Ethics.

[5] Parker WF (2021). Caring for the unvaccinated. Ann Am Thorac Soc.

[6] Coggon J. Should treatments for covid-19 be denied to people who have refused to be vaccinated? [Internet]. The bmg opinion blog; 2021 August 4 [Cited 2022 November 11]. Available from: https://blogs.bmj.com/bmj/2021/08/04/should-treatmentsfor-covid19-be-denied-to-people-who-have-refused-to-be-vaccinated.

[7] Bourke L, Cunningham M, Tomazin F. Doctors' union says medics will treat unvaccinated COVID patients [Internet]. The Sydney Morning Herald; 2021 October 22 [Cited 2022 November 11]. Available from: https://www.smh.com.au/politics/federal/doctorsunion-saysmedics-will-treat-unvaccinated-people-with-covid-20211021-p59263.html.

[8] Schuman O, Robertson-Preidler J, Bibler TM (2022). COVID-19 vaccination status should not be used in triage tie-breaking. J Med Ethics.

[9] New York State Task Force on Life and the Law and New York State Department of Health. Ventilator allocation guidelines [Internet]. 2015 November [Cited 2022 November 11]. Available from: https://www.health.ny.gov/reguations/task_force/reports_publications/docs/ventilator_guidelines.pdf.

[10] Klitzman RL (2020). Legal immunity for physicians during the COVID-19 pandemic: needs to address legal and ethical challenges. Chest.

[11] Dugdale LS. The doctor will see you now—wait not you [Internet]. The Wall Street Journal; 2022 February 8 [Cited 2022 November 11]. Available from: https://www.wsj.com/articles/doctor-unvaccinated-physician-icu-intensivecareunitantivaxxer-refusing-treatment-patients-covid-omicron-immunity-11644336402.

[12] Lieber D. North Texas doctor’s group retreats on policy saying vaccination status to be part of care decisions [Internet]. The Dallas Morning News; 2020 August 19 [Cited 2022 November 11]. Available from: https://www.dallasnews.com/news/watchdog/2021/08/19/ifnorth-texas-runs-out-of-icuhospital-beds-doctors-can-consider-a-patients-vaccination-status/.

[13] Marcus R. Doctors should be allowed to give priority to vaccinated patients when resources are scarce [Internet]. The Washington Post; 2021 September 3 [Cited 2022 November 11]. Available from: https://www.washingtonpost.com/opinions/2021/09/03/doctors-should-be-allowed-give-priority-vaccinated-patients-when-resources-are-scarce/.

[14] Caplan AL. It’s okay for docs to refuse to treat unvaccinated patients [Internet]. Medscape; 2021 October 13 [Cited 2022 November 11]. Available from: https://www.medscape.com/viewarticle/958434.

[15] Andrejko KL, Pry JM, Myers JF, Fukui N, DeGuzman JL, Openshaw J (2022). Effectiveness of face mask or respirator use in indoor public settings for prevention of SARS-CoV-2 infection California, February–December 2021. MMWR Morb Mortal Wkly Rep.

[16] U.S. Food & Drug Administration. N95 Respirators, surgical masks, face masks, and barrier face coverings [Internet]. Updated 2022 August 26 [Cited 2022 November 11]. Available from: https://www.fda.gov/medical-devices/personal-protective-equipment-infection-control/n95-respirators-surgical-masks-face-masks-and-barrier-face-coverings.

[17] English DC (2005). Moral obligations of patients: a clinical view. J Med Philos.

[18] Meyer MJ (1992). Patients' duties. J Med Philos.

[19] Parsons T. The sick role and the role of the physician reconsidered. Milbank Mem Fund Q Health Soc. Summer 1975;53(3):257–78. 10.2307/3349493. 1041510

[20] Ramnath V. Op-Ed: Anti-vaccine patients vent anger on healthcare workers like me. It takes a toll on care [Internet]. Los Angeles Times; 2022 January 20 [Cited 2022 November 11]. Available from: https://www.latimes.com/opinion/story/2022-01-20/unvaccinated-covid-patients-healthcare-workers-turnover-burnout.

[21] Kates J, Tolbert J, Orgera K. The red/blue divide in COVID-19 vaccination rates [Internet]. KFF; 21 September 2021 [Accessed 2022 November 11]. Available from: https://www.kff.org/policy-watch/the-red-blue-divide-in-covid-19-vaccination-rates/.

[22] Kreidle M. No major religious denomination opposes vaccination, but religious exemptions may still complicate mandates [Internet]. Kaiser Family News; 2021 September 9 [Cited 2022 November 11]. Available from: https://www.cnn.com/2021/09/09/health/covid-vaccine-religious-exemptions-khn/index.html.

[23] Pullella P. Pope backs COVID immunisation campaigns, warns of ideological misinformation [Internet]. Reuters; 2022 January 10 [Cited 2022 November 11]. Available from: https://www.reuters.com/world/europe/pope-backs-covid-immunisation-campaigns-warns-ideological-misinformation-2022-01-10/.

[24] Hoonpongsimanont W, Sahota PK, Chen Y, Patel M, Tarapan T, Bengiamin D (2018). Physician professionalism: definition from a generation perspective. Int J Med Educ.

[25] Cruess RL, Cruess SR (2020). Professionalism, communities of practice, and medicine's social contract. J Am Board Fam Med.

[26] American Transplant Foundation. Coronavirus (COVID-19) and transplant patients [Internet]. Last updated 2021 September 13 [Cited 2022 November 11]. Available from: https://www.americantransplantfoundation.org/coronavirus/.

[27] Jameton A (1964). Nursing practice: the ethical issues.

[28] Kälvemark S, Höglund AT, Hansson MG, Westerholm P, Arnetz B (2004). Living with conflicts ethical dilemmas and moral distress in the health care system. Soc Sci Med.

[29] Burston AS, Tuckett AG (2013). Moral distress in nursing: contributing factors, outcomes and interventions. Nurs Ethics.

[30] Fayyad A. The LGBTQ health clinic that faced a dark truth about the AIDS crisis: America has rarely treated all people with HIV equally [Internet]. The Atlantic; 2019 July 22 [Cited 2022 November 11]. Available from: https://www.theatlantic.com/health/archive/2019/07/us-aids-policy-lingering-epidemic/594445/

[31] Shanafelt TD, West CP, Dyrbye LN, Wang H, Carlasare LE, Sinsky C, et al. Changes in burnout and satisfaction with work-life integration in physicians over the first 2 years of the COVID-19 pandemic. Mayo Clin Proc. 2022 September 14. 10.1016/j.mayocp.2022.09.002. 10.1016/j.mayocp.2022.09.002 PMC9472795 36229269

[32] World Health Organization. Shortage of personal protective equipment endangering health workers worldwide [Internet]. 2020 March 3 with undated update [Cited 2022 November 11]. Available from: https://www.who.int/news/item/03-03-2020-shortage-of-personalprotective-equipment-endangering-health-workers-worldwide.

[33] Klitzman R (2007). When doctors become patients.

[34] Shanafelt T, Boone S, Tan L, Dyrbye LN, Sotile W, Satele D (2012). Burnout and satisfaction with work-life balance among US physicians relative to the general US population. Arch Intern Med.

[35] McLernon LM. COVID-related nursing shortages hit hospitals nationwide [Internet]. University of Minnesota CIDRAP; 2020 November 30 [Cited 2022 November 11]. Available from: https://www.cidrap.umn.edu/news-perspective/2020/11/covid-related-nursing-shortages-hit-hospitals-nationwide.

[36] Anders RL (2021). Patient safety time for federally mandated registered nurse to patient ratios. Nurs Forum.

[37] Wan W. Burned out by the pandemic, 3 in 10 health-care workers consider leaving the profession [Internet]. The Washington Post; 2021 April 22 [Cited 2022 November 11. Available from: https://www.washingtonpost.com/health/2021/04/22/health-workers-covid-quit/.

[38] Melnikow J, Padovani A, Miller M (2022). Frontline physician burnout during the COVID-19 pandemic: national survey findings. BMC Health Serv Res.

[39] Norman SB, Feingold JH, Kaye-Kauderer H, Kaplan CA, Hurtado A, Kachadourian L (2021). Moral distress in frontline healthcare workers in the initial epicenter of the COVID-19 pandemic in the United States: Relationship to PTSD symptoms, burnout, and psychosocial functioning. Depress Anxiety.

[40] The Center for Systems Science and Engineering (CSSE) at Johns Hopkins University. COVID-19 dashboard [Internet]. Johns Hopkins University of Medicine Coronavirus Resource Center; Updated 2022 November 11 [Cited 2022 November 11]. Available from: https://coronavirus.jhu.edu/map.html.

[41] Centers for Disease Control and Prevention. COVID Data Tracker [Internet]. CDC; 2022 November 11 [Cited 2022 November 11. Available from: https://covid.cdc.gov/covid-data-tracker/#datatracker-home.

[42] Stephenson J (2021). Costs of preventable COVID-19 hospitalizations during summer surge exceed $5 billion. JAMA Health Forum.

[43] Kaplan CC, Chan CC, Feingold JH, Kaye-Kauderer H, Pietrzak RH, Peccoralo L (2021). Psychological consequences among residents and fellows during the COVID-19 pandemic in New York City: complications for targeted interventions. Acad Med.

[44] Corpora M, Leone AF, Liggett E (2021). Burnout prevention pilot intervention for healthcare workers during COVID-19. J Emerg Manag.

[45] Saddawi-Konefka D, Brown A, Eisenhart I, Hicks K, Barrett E. Gold JA. Consistency between state medical licenseapplications and recommendations regarding physician mental health. JAMA. 2021;325(19):2017–18. 10.1001/jama.2021.2275. 10.1001/jama.2021.2275 PMC8132137 34003231

[46] Hayes JA, Gelso CJ, Goldberg S, Kivlighan DM (2018). Countertransference management and effective psychotherapy: meta-analytic findings. Psychotherapy (Chic).

[47] U.S. House of Representatives. *H.R.1667—Dr. Lorna Breen Health Care Provider Protection Act* [Internet]. openstates; Signed 2022 18 March [Cited 2022 November 11]. Available from: https://www.congress.gov/bill/117th-congress/house-bill/1667/.

[48] Association of American Medical Colleges (AAMC). AAMC endorses bill promoting physician mental health and well-being [Internet]. AAMC; 2021 March 12 [Cited 2022 November 11]. Available from: https://www.aamc.org/advocacy-policy/washington-highlights/aamc-endorses-bill-promoting-physician-mental-health-and-well-being.

[49] Brown SD, Goske MJ, Johnson CM. Beyond substance abuse: stress, burnout, and depression as causes of physician impairment and disruptive behavior. J Am Coll Radiol. 6(7);479–85. 10.1016/j.jacr.2008.11.029. 10.1016/j.jacr.2008.11.029 19560063

[50] Aaronson AL, Backes K, Agarwal G, Goldstein JL, Anzia J (2018). Mental health during residency training: assessing the barriers to seeking care. Acad Psychiatry.

[51] Saddawi-Konefka D, Brown A, Eisenhart I, Hicks K, Barrett E, Gold JA (2021). Consistency between state medical license applications and recommendations regarding physician mental health. JAMA.

[52] Dyrbye LN, West CP, Sinsky CA, Goeders LE, Satele DV, Shanafelt TD (2017). Medical licensure questions and physician reluctance to seek care for mental health conditions. Mayo Clin Proc.

[53] National Academies of Sciences, Engineering, and Medicine; National Academy of Medicine; Committee on Systems Approaches to Improve Patient Care by Supporting Clinician Well-Being. Taking action against clinician burnout: a systems approach to professional well-being. Washington DC: National Academies Press (US); 2019 Oct 23. 10.17226/25521. 31940160

